# Waist Circumference and All-Cause Mortality Independent of Body Mass Index in Korean Population from the National Health Insurance Health Checkup 2009–2015

**DOI:** 10.3390/jcm8010072

**Published:** 2019-01-10

**Authors:** Yang-Hyun Kim, Seon Mee Kim, Kyung-Do Han, Jin-Hyung Jung, Seong-Su Lee, Sang Woo Oh, Hye Soon Park, Eun-Jung Rhee, Won-Young Lee, Soon Jib Yoo

**Affiliations:** 1 Department of Family Medicine, Korea University College of Medicine, Seoul 02841, Korea; mrchir@naver.com (Y.-H.K.); ksmpdh@korea.ac.kr (S.M.K.); 2 Department of Medical Statistics, Catholic University College of Medicine, Seoul 06591, Korea; hkd917@naver.com (K.-D.H.); jungjin115@naver.com (J.-H.J.); 3 Division of Endocrinology and Metabolism, Department of Internal Medicine, The Catholic University of Korea, Sosa-ro 327, Wonmi-gu, Bucheon 14647, Korea; mddaniel@catholic.ac.kr; 4 Department of Family Medicine, Center for Obesity, Metabolism and Nutrition, Dongguk University Ilsan Hospital, Dongguk University College of Medicine, Goyang 10326, Korea; osw6021@gmail.com; 5 Department of Family Medicine, Ulsan University College of Medicine, Seoul 05505, Korea; hyesoon@amc.seoul.kr; 6 Division of Endocrinology and Metabolism, Department of Internal Medicine, Kangbuk Samsung Hospital, Sungkyunkwan University School of Medicine, Seoul 03181, Korea; hongsiri@hanmail.net

**Keywords:** waist circumference, body mass index, obesity, mortality, Korean National Health Insurance Corporation

## Abstract

Background: Waist circumference (WC) is an index of abdominal obesity and associated with co-morbidities and mortality. Higher WC is positively associated with increased mortality; therefore, we examined the relationship between WC and mortality in Korean populations with the interaction of body mass index (BMI) and WC for mortality. Methods: A total of 23,263,878 subjects (men = 11,813,850 and women = 11,450,028) who were older than 20 years and underwent the National Health Insurance Service health checkup were included. WC was divided into six categories by 5 cm increments and level 3 (85–90 cm in men and 80–85 cm in women) was referenced. Multivariable Cox proportional hazard models were used to obtain the hazard ratios (HRs) and 95% confidence intervals for all-cause mortality according to the six levels of WC. Results: WC in 5 cm increments showed a positively increased all-cause mortality after adjusting for all covariates including BMI. Men showed higher HRs for mortality than women as WC increased, and the HRs were higher in the lower WC levels, but lower in the higher WC levels among the subjects aged 65–85 years than subjects aged 40–65 years. Even in subjects with normal weight and overweight, increased WC (levels 4, 5, and 6) showed increased HRs for mortality (HRs = 1.156, 1.412, and 1.614 in normal BMI and 1.145, 1.401, and 1.909 in overweight, respectively). Conclusion: There was a linear association between WC and all-cause mortality across all BMI categories even in the subjects with normal or overweight BMI. Physicians should check WC routinely even in the subjects with normal weight or overweight.

## 1. Introduction

Obesity is a status of excessive body fat and defined as a body mass index (BMI) of more than 30 kg/m^2^ in the United States, or 25 kg/m^2^ in Asian countries including South Korea [1,2]. Obesity is one of the major risk factors for co-morbidities and mortality [3,4], and in two Korean studies, BMI was associated with reverse J shaped all-cause mortality curves [5,6]. However, BMI itself has limitations in explaining the relationship between obesity and morbidity or mortality because BMI does not account for the exact fat mass, especially visceral adipose tissue [3,7,8].

Waist circumference (WC) is an index for abdominal obesity [9] and has been known as a better predictor than BMI for the prevalence or incidence of co-morbidities such as type 2 diabetes mellitus (DM) [10,11,12], hypertension [12], dyslipidemia [12,13], and coronary heart diseases [14]. Many studies have shown that a larger WC is also associated with increased mortality although the strength of the association varies among studies [8,15,16,17,18,19,20,21,22,23,24]. Moreover, many studies have shown this association even after adjusting for BMI [17,20,22,23,24]. Even in a pooled analysis, higher WC was positively associated with increased mortality in white adults at BMI levels between 20 and 50 kg/m^2^ [25]. These studies were performed mostly in Europe [8,16,17,20], the United States [19,20,21,22,23,24,26], or among Chinese women [27]; however, there was a lack of evidence in the association between WC and mortality in Korean populations. Therefore, we examined the relationship between WC and all-cause mortality using data from the National Health Insurance Service (NHIS) health checkup.

## 2. Methods

### 2.1. The NHIS Database and NHIS Health Checkup Data

The NHIS manages the NHI program including about 50 million of the Korean population and their medical information. This medical information consists of patients’ age, sex, living area, insurer payment coverage, deduction and claims data, and medical utilization/transaction information. The Health Insurance Review and Assessment (HIRA) database contains health insurance claims data of 97.0% of the Korean population and the HIRA service has the source population of the NHIS [28,29]. All insured Koreans older than 40 years undergo a biannual health checkup supported by the NHIS, and employees older than 20 years are required to undergo health checkup once a year. In the NHI health checkup, weight (kg), height (cm), WC (cm), systolic blood pressure (mmHg), and diastolic blood pressure (mmHg) are measured. Also, fasting blood glucose (mg/dL), total cholesterol (mg/dL), triglyceride (mg/dL), high-density lipoprotein cholesterol (mg/dL), low-density lipoprotein cholesterol (mg/dL) levels are also measured in a fasting state. General health behaviors such as alcohol drinking, smoking, and exercise are surveyed, and past medical history of cancer, tuberculosis, and chronic diseases such as type 2 DM, HTN, and dyslipidemia are obtained by self-reported questionnaires. The quality of the laboratory tests are warranted by the Korean Association for Laboratory Medicine, and the NHIS certifies the hospitals participating in the NHI health checkup programs [30]. In this study, we used the NHI health checkup database from 2009 to 2015.

#### Subjects

From the NHI health checkup between 2009 and 2015, a total of 23,503,802 participants older than 20 years were included. These subjects were followed-up to 31 December 2015. We excluded subjects with missing data (*n* = 125,699; 0.5348%), those younger than 20 and older than 85 (*n* = 114,209), and subjects with unmatched mortality information (*n* = 16) over four consecutive years. Finally, 23,263,878 subjects were included in this study (men = 11,813,850 and women = 11,450,028). Subjects aged more than 85 during follow up were 145,353 (0.62%). The study protocol was approved by the Institutional Review Board of the Korea University Anam Hospital (ED-17115) and permission for the use of health checkup data was granted by the NHIS (NHIS-2018-1-035).

### 2.2. Definition of Obesity and Abdominal Obesity

We defined obesity as a BMI ≥25 kg/m^2^ and categorized it into underweight (BMI < 18.5 kg/m^2^), normal (18.5 ≤ BMI < 23 kg/m^2^), overweight (23 ≤ BMI < 25 kg/m^2^), stage I obesity (25 ≤ BMI < 30 kg/m^2^), and stage 2 obesity (BMI ≥ 30 kg/m^2^) according to the World Health Organization recommendations for Asians [1]. Abdominal obesity was defined as WC ≥ 90 cm in men and ≥ 85 cm in women according to the definition of the Korean Society for the Study of Obesity [31]. WC was divided into six categories with increments of 5 cm—level 1: <80 cm in men and <75 cm in women, level 2: 80–85 cm in men and 75–80 cm in women, level 3: 85–90 cm in men and 80–85 cm in women, level 4: 90–95 cm in men and 85–90 cm in women, level 5: 95–100 cm in men and 90–95 cm in women, and level 6: ≥100 cm in men and ≥95 cm in women.

### 2.3. General Health Behavior and Socio-Demographic Variables

Subjects were also categorized according to smoking status: non-smokers, former smokers, or current smokers. Alcohol drinking was categorized into none, moderate, or heavy drinkers (≥3 days/week) and regular exercise defined as vigorous physical activity for at least 20 min/day. Income was divided by quartile (Q): Q1 (the lowest), Q2, Q3, and Q4 (the highest). Residence in urban areas was also checked.

### 2.4. All-Cause Mortality

All-cause mortality was checked between 1 January 2009 and 31 December 2015 for each participant, and the number of person-years of follow-up was also counted. All-cause mortality was assessed within 5.39 ± 1.11 years after the last recorded WC value and there were a total of 502,456 deaths.

### 2.5. Statistical Analysis

The general characteristics of subjects are presented as means ± standard deviation (SD) for continuous variables and percentages (SD) for categorical variables, according to the six levels of WC. The hazard ratios (HRs) and 95% confidence intervals (CIs) for all-cause mortality according to the six levels of WC were analyzed using multivariable Cox proportional hazard models, using the level 3 WC (85–90 cm in men and 80–85 cm in women) as a reference group, after adjusting for age and sex in model 1; age, sex, smoking, alcohol drinking, regular exercise, and income (Q1) in model 2; and all covariates of model 2 and BMI in model 3. We did not adjust for chronic disease such as DM, hypertension, and dyslipidemia to avoid bias because such chronic diseases are believed to be on the causal pathway from BMI to mortality [32]. The HR and 95% CI for all-cause mortality according to the six levels of WC were also obtained using a multivariable Cox model for the different subgroups: sex, three age groups (20–40 years, 40–64 years, and 65–84 years) after adjusting for all covariates. We also obtained HRs and 95% CI for all-cause mortality of WC levels regarding level 3 WC as the reference level in the five BMI groups (<18.5 kg/m^2^, 18.5–23 kg/m^2^, 23–25 kg/m^2^, 25–30 kg/m^2^, and ≥30 kg/m^2^). All statistical analyses were performed using SAS version 9.3 (SAS Institute Inc., Cary, NC, USA), and *p* < 0.05 for two-tailed *t*-tests was considered statistically significant.

## 3. Results

Table 1 shows the general characteristics of participants by the six levels of WC. As WC level increases, age and BMI 25–30 kg/m^2^ increased until level 5 of WC. The proportion of the 65–84 year age group increased. BMI and BMI ≥ 30 kg/m^2^ increased as WC increased. The proportion of non-smokers, non-alcohol drinkers, and the lowest income level (Q1) showed a U-shaped curve as WC increased. The mortality rate increased as WC increased.

The adjusted HRs for all-cause mortality are shown in Table 2 and supplement Figure S1 according to the six WC groups. WC and mortality showed a reverse J shaped curve before adjusting for BMI in model 2, but showed a positive linear association between WC and all-cause mortality after adjusting for BMI in model 3. We also analyzed the association between all-cause mortality and WC according to risk factors such as smoking, alcohol drinking, regular exercise, and income level in our unpublished study. The HRs for all-cause mortality also increased linearly as WC level increased and there was no additive effect on all-cause mortality by risk factors.

**Figure 1:**
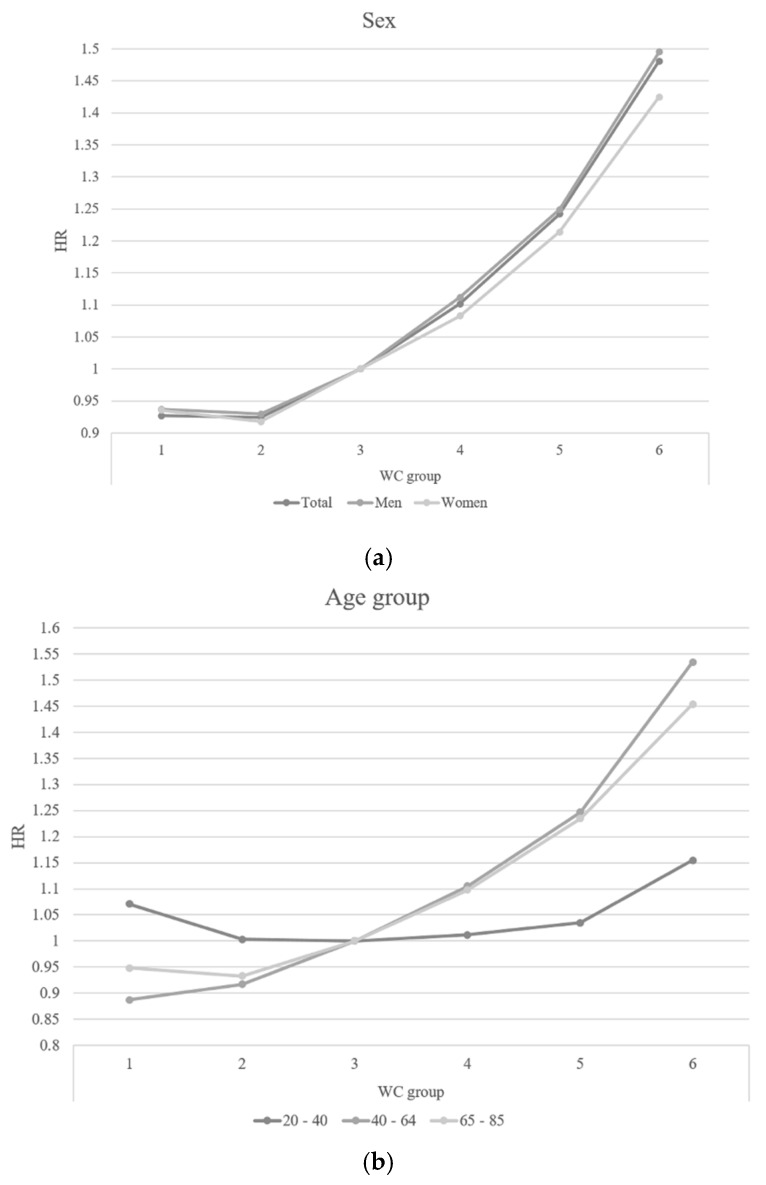
Hazard ratio for all-cause mortality by sex and age group in WC groups. (**a**) Sex: Adjusted for age, sex, smoking, drinking, exercise, income, and body mass index; WC: waist circumference, HR: hazard ratio, C.I.: confidence interval (**b**) Age group: Adjusted for age, sex, smoking, drinking, exercise, income, and body mass index; WC: waist circumference, HR: hazard ratio, C.I.: confidence interval.

**Figure 2:**
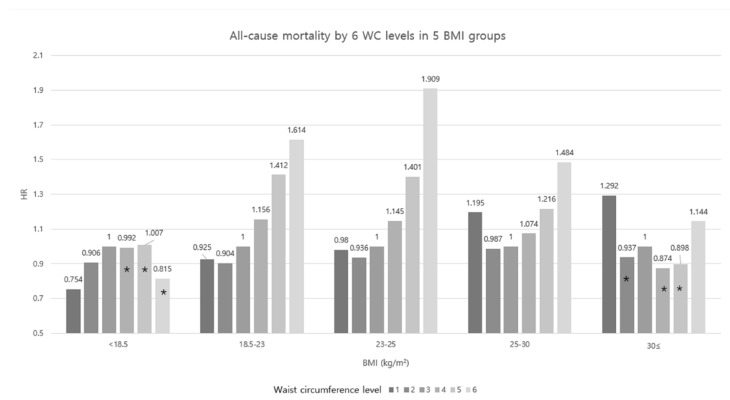
All-cause mortality by waist circumference and BMI. Adjusted for age, sex, smoking, drinking, exercise, income, and body mass index. WC: waist circumference, HR: hazard ratio, and BMI: body mass index. * Statistically not significant.

Figure 1 shows the subgroup analysis in the relationship between WC and mortality according to sex and three age groups. After adjusting for all covariates, both men and women showed a linear association between WC and all-cause mortality and men showed higher HRs for mortality than women (Figure 1a). The age group 20–40 years showed a weak association between WC and mortality, but both 40–65 and 65–85 year age groups showed positive linear associations between WC and mortality. In the level 1 and 2 WC categories, the HRs for mortality were higher for ages 65–85 years than the ages 40–65 years, but in levels 4, 5, and 6, the HRs for mortality were higher for ages 40–65 years than the ages of 65–85 years (Figure 1 and Figure 2).

Figure 2 shows the WC and mortality in the five BMI categories. When WC level 3 was regarded as the reference group, lower levels of WC (levels 1 and 2) showed lower HRs for mortality than level 3 in underweight BMI, but higher levels of WC groups did not show any associations. In subjects with normal weight, overweight, and stage 1 obesity, HRs for mortality increased as WC increased. In WC levels 4 and 5, the HRs were the highest in the normal BMI than overweight and stage 1 obesity BMI (HRs = 1.156 and 1.412 in normal BMI, respectively). In level 6 WC, the HR was the highest in overweight BMI than the normal weight and stage 1 obesity (HRs = 1.909, 1.614, and 1.484, respectively). However, in stage 2 obesity, level 1 WC showed higher HR (HR = 1.292) than level 6 WC (HR = 1.144) and the association between WC and mortality were not significant in the other levels of WC.

## 4. Discussion

In this study, 5 cm increments in WC showed increasing all-cause mortality after adjusting for all covariates independent of BMI. Men showed higher HRs for mortality than women as WC increased, and the HRs were higher in the lower WC levels, but lower in higher WC levels in the subjects with age 65–85 years than subjects aged 40–65 years. Even in subjects with normal weight, higher WC levels showed higher HRs for mortality than lower WC levels, and this association was also found in overweight and stage I obesity.

Our results are consistent with previous studies which showed that higher WC was associated with higher risk for mortality even after adjusting for BMI [17,20,22,24,26,27]. In a large US cohort study, 10 cm increases in WC was associated with positive increase of all-cause mortality and very high levels of WC (≥120 cm in men and ≥110 cm in women) were associated with about 2-fold higher relative risk for mortality in men and women compared to low levels of WC (<90 cm in men and <75 cm in women) (relative risk = 2.02; 95% CI: 1.71–2.39 in men and 2.36; 95% CI: 1.98–2.82 in women). Women showed higher HRs for all-cause mortality than men at all levels of WC after adjusting for multiple variables including BMI [26].

In this study, even in the subjects with normal BMI and overweight BMI subjects with levels 4, 5, and 6 of WC (abdominal obesity) showed increased risks for mortality than subjects with level 3 WC (15.6%, 41.2%, and 61.4%, respectively in normal BMI and 14.5%, 40.1%, and 90.9% in overweight BMI). In the large National Institutes of Health—American Association of Retired Persons (NIH-AARP) cohort, subjects with normal BMI with abdominal obesity (≥88 cm in women and ≥102 cm in men) had approximately 20% higher risk for mortality than subjects with normal BMI and non-abdominal obesity [24]. In the European Prospective Investigation into Cancer and Nutrition (EPIC) cohort and the Nurses’ Health Study, WC also showed positive associations with mortality even in the normal range BMI (<25 kg/m^2^) [17,24]. In the US population, normal BMI showed more increased HR for WC associated mortality than other BMI categories [26]. In a meta-analysis including 58,609 subjects from 23 studies, higher WC was also associated with higher mortality in the elderly subjects with both normal and overweight BMI [33]. However, in our study, overweight BMI showed the stronger association between WC and mortality than normal BMI. This different result indicates that there may be ethnic differences between WC and mortality and WC is more meaningful in the overweight BMI categories than normal or obesity BMI categories in Koreans. These results mean that we should assess the distribution of body fat especially in the study of mortality and there may be a need to decrease abdominal fat to reduce mortality especially in subjects with normal or overweight BMI [2].

Men had higher HR for mortality than women in all WC levels in this study. In the EPIC studies, 5 cm increase of WC was associated with 17% increase of mortality risk in men and 13% increase of mortality risk in women [17]. Men had more visceral fat than subcutaneous fat in the same WC than those of women [34], so men with higher WC may show more increased mortality than women. However, the exact mechanism is unclear. More detailed designed studies regarding these sex differences are needed in the future.

In a US population study, relative risks for mortality associated with WC were lower in men younger than 70 years than in those more than 70 years [26]. The different effect of WC in the different age groups is unclear, but, higher mortality in lower WC levels is maybe explained by low BMI, which is associated with higher mortality [3] and a weaker association between WC and the volume of visceral fat among younger population than older population [35].

The mechanism between increased WC and increased mortality can be explained by the effect of visceral adipose tissue. Higher WC is associated with higher visceral adipose tissue [36,37] which is more pathogenic than subcutaneous tissue [36,38]. Visceral adipose tissue secretes some mediators which may develop cardiometabolic diseases which increases the risk of death in the subjects with higher WC [39]. Moreover, increased WC is associated with and inflammation [40,41], insulin resistance [42,43], type 2 DM [10,11], and coronary heart diseases [14,44], which all are associated with increased mortality. After adjusting for BMI, the linearity and strength of association between WC and mortality were found. This may due to a decrease of confounding factors such as pathologic conditions or pre-existing comorbidities, or frailty, which are all associated with low BMI [45].

This study has several limitations. First, we do not know the cause of death. Some cancers or respiratory diseases such as chronic obstructive pulmonary diseases decrease weight and then WC also may decrease. So, low WC may be associated with increased mortality contrary to this study. Second, we only checked WC at one time when study involved, so we could not know the status of WC whether WC has been decreasing or increasing. Third, we do not have body composition data, so we could not know that increased mortality was associated with increased visceral fat or muscle wasting. Fourth, Fourth, the time of follow-up to mortality after anthropomorphic measurements were taken is too short. Fifth, we calculated the HR by adding covariates, but a HR is a non-collapsible measure, so we should use a population averaging method to correct for this. We added age and sex standardized incidence rate based on Korea 2010 Census instead of incidence rate in supplement Table S1.

However, this study has some strengths. As we know, this is the first study to examine the relationship between WC and all-cause mortality in the large Korean population. Second, some studies assessed WC by participants’ memory, but we measured all participants’ WC by trained examiners in certified facilities. Third, this study included 502,456 deaths, which is more than the number of deaths from the EPIC study and NIH-AARP cohort study [17,23]. Fourth, we adjusted for variable covariates which potentially affect mortality.

## 5. Conclusions

In conclusion, our large cohort study found a linear association between WC and all-cause mortality across the full range of BMI categories and this association was also found in the subjects with normal or overweight BMI. Several guidelines including the National Health Institute guidelines only suggested the loose of weight or WC in the obese subjects [9], but this study provides evidence to the necessity for losing WC in the subjects with normal or overweight BMI categories. Moreover, WC is not routinely measured in the non-obese subjects or older subjects. Physicians are better to check WC routinely especially in the subjects with high WC categories and the guidelines specifying acceptable WC standard would benefit the Korean public. Further detailed designed studies are needed to evaluate the cause of death be increased WC and the mechanism of this association.

## Figures and Tables

**Table 1:** General characteristics of participants by waist circumference.

	Waist Circumference Group					
	1	2	3	4	5	6
*N*	8,672,967	5,362,342	4,538,812	2,714,222	1,253,456	722,079
Men (yes,%)	3,450,338(39.78)	3,079,604(57.43)	2,648,218(58.35)	1,604,864(59.13)	679,425(54.2)	351,401(48.67)
Age (year)	42.69 ± 13.76	48.24 ± 13.42	50.91 ± 13.44	52.43 ± 13.67	53.33 ± 14.18	52.5 ± 15.11
Age group (yes,%)						
20–40	3,596,630(41.47)	1,384,164(25.81)	915,952(20.18)	492,555(18.15)	225,771(18.01)	158,788(21.99)
40–64	4,399,758(50.73)	3,297,574(61.5)	2,842,275(62.62)	1,651,324(60.84)	720,342(57.47)	381,554(52.84)
65–85	676,579(7.8)	680,604(12.69)	780,585(17.2)	570,343(21.01)	307,343(24.52)	181,737(25.17)
Body mass index (kg/m^2^)	21.05 ± 2.04	23.51 ± 1.89	24.99 ± 2.01	26.47 ± 2.16	28.04 ± 2.37	30.68 ± 3.21
<18.5	901,949(10.4)	24,771(0.46)	6702(0.15)	2197(0.08)	638(0.05)	287(0.04)
18.5–23	6,323,446(72.91)	2,090,324(38.98)	678,158(14.94)	125,309(4.62)	20,215(1.61)	5471(0.76)
23–25	1,213,690(13.99)	2,156,699(40.22)	1,637,121(36.07)	516,685(19.04)	90,541(7.22)	14,870(2.06)
25–30	230,364(2.66)	1,080,974(20.16)	2,167,188(47.75)	1,925,391(70.94)	897,990(71.64)	288,858(40)
≥30	3518(0.04)	9574(0.18)	49,643(1.09)	144,640(5.33)	244,072(19.47)	412,593(57.14)
Smoking (yes,%)						
Non	5,964,846(68.78)	3,106,503(57.93)	2,592,749(57.12)	1,531,141(56.41)	741,549(59.16)	446,954(61.9)
Former	786,403(9.07)	812,076(15.14)	765,533(16.87)	481,828(17.75)	205,172(16.37)	100,249(13.88)
Current	1,921,718(22.16)	1,443,763(26.92)	1,180,530(26.01)	701,253(25.84)	306,735(24.47)	174,876(24.22)
Alcohol drinking (yes,%)						
Non	4,784,499(55.17)	2,741,740(51.13)	2,368,663(52.19)	1,433,874(52.83)	700,834(55.91)	425,314(58.9)
Moderate	3,422,946(39.47)	2,178,086(40.62)	1,756,031(38.69)	1,006,673(37.09)	426,554(34.03)	225,813(31.27)
Heavy	465,522(5.37)	442,516(8.25)	414,118(9.12)	273,675(10.08)	126,068(10.06)	70,952(9.83)
Regular exercise (yes,%)	1,405,021(16.2)	1,027,051(19.15)	864,575(19.05)	498,760(18.38)	216,517(17.27)	112,544(15.59)
Income (Q1) (yes,%)	1,931,941(22.28)	1,090,911(20.34)	911,431(20.08)	551,669(20.33)	265,490(21.18)	163,770(22.68)
Urban living (yes,%)	4,058,601(46.82)	2,471,135(46.11)	2,065,373(45.53)	1,211,852(44.68)	549,845(43.91)	31,2001(43.27)
Death (yes,%)	149,733(1.73)	111,307(2.08)	106,771(2.35)	72,606(2.68)	37,763(3.01)	24,276(3.36)

**Table 2:** Waist circumference and all-cause mortality.

					HR (95% C.I.)		
WC Group	*N*	Death	Duration	Incidence Rate	Model 1	Model 2	Model 3
1	8,672,967	149,733	46,394,946.4	3.22736	1.375(1.364,1.386)	1.328(1.317,1.338)	0.927(0.919,0.936)
2	5,362,342	111,307	29,089,043.6	3.82642	1.069(1.06,1.078)	1.06(1.051,1.069)	0.925(0.917,0.933)
3	4,538,812	106,771	24,633,745.2	4.33434	1	1	1
4	2,714,222	72,606	14,677,575.8	4.94673	0.993(0.984,1.003)	0.995(0.985,1.004)	1.102(1.091,1.113)
5	1,253,456	37,763	6,731,707.3	5.60972	1.063(1.051,1.076)	1.061(1.048,1.073)	1.242(1.226,1.257)
6	722,079	24,276	3,814,427.1	6.36426	1.262(1.245,1.28)	1.247(1.23,1.264)	1.481(1.457,1.505)

Model 1 was adjusted for age and sex; Model 2 was adjusted for age, sex, smoking, drinking, exercise, and income.; Model 3 was adjusted for age, sex, smoking, drinking, exercise, income, and body mass index; WC; waist circumference, HR; hazard ratio, and C.I.; confidence interval.

## Supplementary Materials

The following are available online at http://www.mdpi.com/2077-0383/8/1/72/s1, Figure S1: Smoothed hazard ratio for waist circumference; Table S1: Age and sex standardized incidence rate based on Korea 2010 Census.
