# Supplementary material for: Waist Circumference and All-Cause Mortality Independent of Body Mass Index in Korean Population from the National Health Insurance Health Checkup 2009–2015
Source: J Clin Med. 2019 Jan 10;8(1):72. doi: 10.3390/jcm8010072 (PMC6352259; doi:10.3390/jcm8010072)
Supplement: Supplementary file 1 [file jcm-08-00072-s001.pdf]

Journal of
Clinical Medicine

Supplement figure 1. Smoothed hazard ratio for waist circumference

1) Before adjusting for body mass index

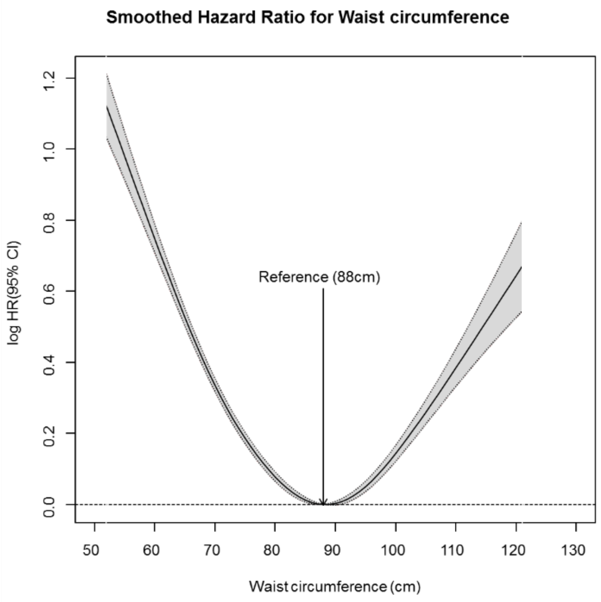

Adjusted for age, sex, smoking, drinking, exercise, and income

HR; hazards ratio, and C.I.; confidence interval

2) After adjusting for body mass index

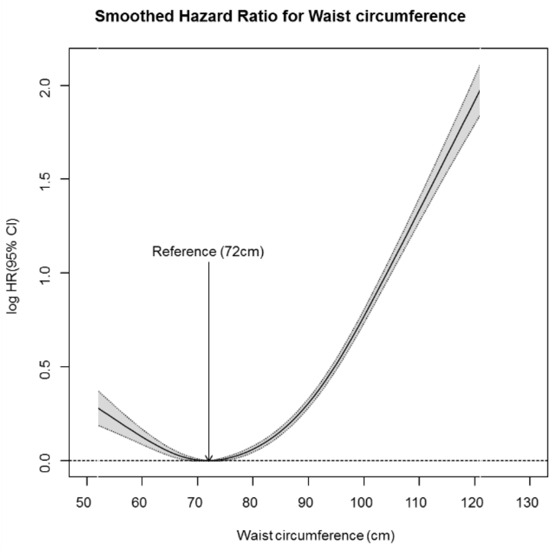

Adjusted for age, sex, smoking, drinking, exercise, income, and body mass index

HR; hazards ratio, and C.I.; confidence interval

Supplement table 1. Age and sex standardized incidence rate based on Korea 2010 Census

WC group	N	Death	Duration	aIR*	Lower	Upper
1	8672967	149733	46394946.4	5.10	5.07	5.13
2	5362342	111307	29089043.6	4.03	4.00	4.05
3	4538812	106771	24633745.2	3.79	3.76	3.81
4	2714222	72606	14677575.8	3.75	3.72	3.78
5	1253456	37763	6731707.3	3.97	3.93	4.02
6	722079	24276	3814427.1	4.62	4.55	4.68

* Age and sex standardized incidence rate
